# Study on Organo-Silica-Derived Membranes Using a Robeson-like Plot

**DOI:** 10.3390/membranes15030083

**Published:** 2025-03-05

**Authors:** Lucas Bünger, Tim van Gestel, Tim Kurtz, Krassimir Garbev, Peter Stemmermann, Wilhelm A. Meulenberg, Olivier Guillon, Dieter Stapf

**Affiliations:** 1 Institute for Technical Chemistry, Karlsruhe Institute of Technology, 76344 Karlsruhe, Germany; tim.kurtz@kit.edu (T.K.); krassimir.garbev@kit.edu (K.G.); dieter.stapf@kit.edu (D.S.); 2 Institute of Energy Materials and Devices-IMD-2, Forschungszentrum Jülich GmbH, 52425 Jülich, Germany; t.van.gestel@fz-juelich.de (T.v.G.); w.a.meulenberg@fz-juelich.de (W.A.M.); o.guillon@fz-juelich.de (O.G.)

**Keywords:** microporous membrane, BTESE, CO_2_ separation, binary mixtures, Robeson-like plot

## Abstract

For industrial CO_2_ utilization, the supply of concentrated CO_2_ within a continuous, high-volume stream at high temperatures remains a substantial requirement. Membrane processes offer a simple and efficient method to provide CO_2_ in this form. While several organo-silica-based membranes have been developed for CO_2_/N_2_ separation under these conditions, there is no standardized framework guiding comparability and optimization. Therefore, we present these membranes in a Robeson-like plot across various temperatures. Utilizing a standard 1,2-bis(triethoxysilyl)-ethane (BTESE) precursor and a simplified sol–gel method, we prepared a microporous membrane layer and characterized it for an exemplary comparison. This characterization includes key parameters for mixed-gas applications: (1) temperature-dependent single- and mixed-gas permeances to observe interactions, (2) the impact of the driving forces in mixtures (vacuum and concentration) to distinguish between permselectivity and the separation factor clearly, and (3) influence of the support structure to enable permeability calculations at elevated temperatures. Furthermore, a quick interpretation method for assessing the membrane’s microstructure is presented. A qualitative microstructure assessment can be achieved by analyzing the temperature dependencies of the three major diffusion mechanisms that simultaneously occur—Knudsen, surface, and activated diffusion.

## 1. Introduction

An important way to compensate for remaining fossil carbon emissions is to capture carbon from unavoidable industrial emissions and reutilize it as a carbon source in chemical processes. According to Favre et al. [1], processes in the industry generate continuous exhaust streams with CO_2_ concentrations between 5 and 30%. Carbon utilization processes, such as the reverse water–gas shift reaction (rWGS), require temperatures above 250 °C, making CO_2_ capture at these temperatures more economical [2]. The state-of-the-art separation methods based on washing and adsorption typically yield CO_2_ discontinuously at low temperatures, which is less efficient for subsequent utilization. Although it is possible to implement intelligent process interconnections to achieve a pseudo-continuous process, like in pressure swing adsorption (PSA), this would require advanced process control and high investment costs [3]. A membrane separation process with a CO_2_-affine membrane appears as an attractive, simple, and straightforward alternative because it enables removing CO_2_ continuously from an industrial emission stream and constantly delivering a concentrated CO_2_ stream for further utilization [4]. Notably, a membrane can be regarded as a continuous PSA application by spatially separating adsorption and desorption through diffusion across the membrane [5].

**Figure 1:**
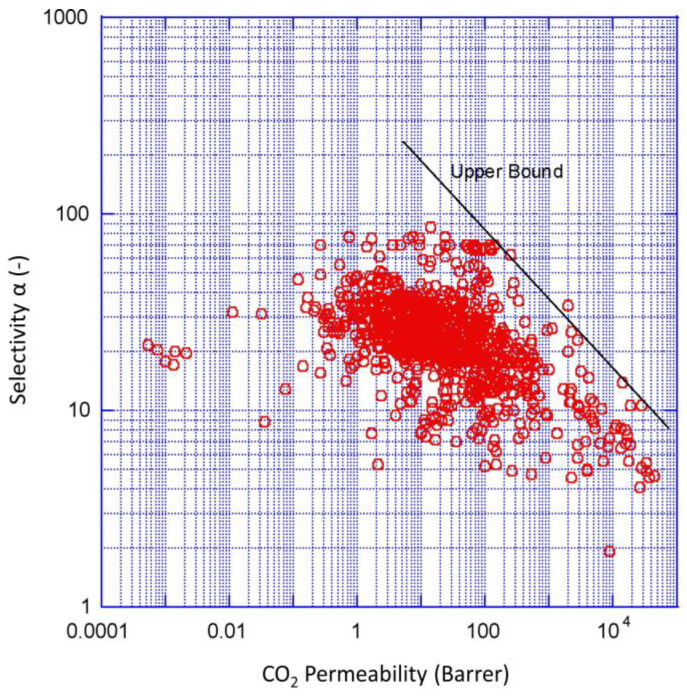
Robeson plot upper bound correlation for CO_2_/N_2_ separation around standard temperature for various polymeric membranes. Reprinted from Robeson, L.M. The upper bound revisited. *J. Membr. Sci*. 2008, 320, 390–400 with permission from Elsevier [7].

In recent years, major progress has been made in gas-separation polymer membranes. New classes of CO_2_-philic polymers have been developed with improved performance for CO_2_ separation and capture [6]. Many polymeric membranes investigated so far are summarized in the Robeson plot in Figure 1, with its upper bound defined by the intrinsic mass transport properties of CO_2_ and N_2_ in polymers [7]. However, for industrial applications, the operating conditions in the industry (e.g., high temperature) present a major challenge for polymer membrane development [8]. There are no Robeson plots for membranes above standard temperature due to the changing membrane properties at high temperatures that render CO_2_ separation impossible.

**Figure 2:**
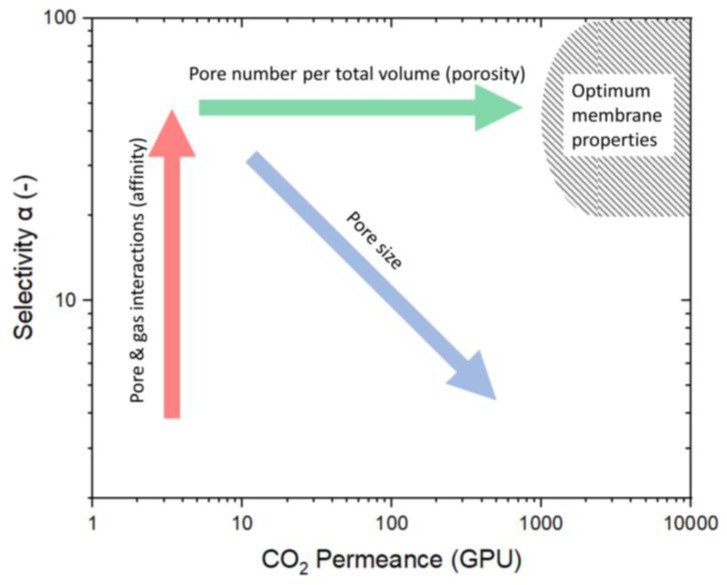
Robeson-like plot for porous materials. This schematic illustrates the ways to influence the membrane design criteria to reach the optimum membrane properties as described by Merkel et al. [9]. The increase in selectivity is predominantly determined by the membrane material (red arrow), whereas the permeance is based on pore size (blue arrow) and pore number (green arrow).

Merkel et al. [9] displayed a variety of porous membranes in a Robeson-like plot, with permeance rather than permeability as the membrane kinetics (Figure 2). Considering the economic suitability, the authors included an area of optimal membrane properties as a target for future developments. Membranes with optimal properties have at least a permeance for CO_2_ above 1000 GPU (GPU = 10^−6^ cm^3^ (STP)/(cm^2^ s cmHg)) and a single-gas selectivity higher than 20. Optimizing membranes to reach the upper boundary may lead to high selectivities but low permeabilities. These membranes have a very low permeability for the unselective component, causing relatively high partial pressures of the selective component in the permeate. As a result, this leads to low fluxes of the selective compound caused by the small partial pressure difference and requires larger membrane areas to separate high-feed streams. Therefore, the (Robeson) upper bound is not the technically relevant limitation, as membranes with low fluxes and high selectivity have no technical relevance in separating gas mixtures [9].

Several design criteria must be met to reach the envisioned optimal membrane properties. Most importantly, the membrane must be made of selective material for the preferred component to permeate with higher kinetics. Differences in the affinity between gas and membrane determine the selectivity (marked by red arrow). For this affinity to become relevant, pores must be below 2 nm, as only these micropores have a favorable surface-to-volume ratio where the flow along the pore surface exceeds the volume flow. However, as the pores become larger, the permeance increases, but the selectivity decreases (marked by the blue arrow in Figure 2). Increasing the pore number is the only way to shift a microstructure with small pores and high selectivity into the envisioned area. In other words, the ratio of the number of micropores to membrane volume (porosity) needs to be high (marked by the green arrow) [10,11].

In designing a membrane to separate CO_2_ from N_2_, it is necessary to make use of the right property difference. The kinetic diameters of CO_2_ (0.33 nm) and N_2_ (0.365 nm) are too close, and a precise pore size would be necessary. The weight of N_2_ (28 g mol^−1^) is smaller than that of CO_2_ (44 g mol^−1^). Therefore, Knudsen-based separation would prefer N_2_ permeation with low selectivity. Thus, only a separation based on their affinity with surfaces is applicable. CO_2_ has a quadrupole that enables adsorption on basic (oxygen) sites [12]. This property enables surface diffusion, which can lead to high selectivities if the microstructure is set appropriately. At first glance, a membrane with ultramicropores would be preferable for CO_2_ separation and purification as high selectivities are possible. However, a small pore size down to the range where molecular sieving dominates is also accompanied by very low flow rates for the non-selective component, demanding very high membrane areas to be technically relevant [9]. Therefore, we opted for a membrane with slightly larger micropores consisting of a CO_2_-philic material. In the literature, various CO_2_ affine materials with a network of connected micropores of approximately 0.5 nm diameter have been reported, including zeolites [13], metal–organic frameworks (MOFs) [14], carbon-based membranes [12], and sol–gel-derived silica [15]. Silica membranes, in particular, have been intensively investigated due to their relatively simple formation by the sol–gel coating process. They are characterized by relatively good selectivity for CO_2_ while maintaining adequate permeability. However, interest in this material has diminished considerably due to concerns about its stability in water (vapor) [16]. An extension of the work on silica membranes is the introduction of hybrid carbon-modified silicas. They are produced similarly to the silica membranes but, with alkane-bridged silicon precursors. The precursor used in this work is the 1,2-bis(triethoxysilyl) ethane (BTESE). This compound is a precursor for silica-bridged aerogels with versatile applications, i.e., in environmental applications in oil/water separation [17] or even in antibiotic adsorption from water [18]. Notably, the organo-silica aerogels showed excellent behavior in gas separation applications [16,19,20,21,22].

Focusing on the temperature-dependent transport behavior can provide insights into the pore structure, extending the work of Lee et al. [23]. A comparison with the reported organo-silica-derived membranes exhibits permeances ranging from as low as 30 GPU [24] to as high as 9500 GPU [25]. Although all the membranes are fabricated similarly, no comprehensive analysis for this wide range of reported kinetics has yet been provided.

As all technical applications aim to operate in mixtures, the mixed-gas permeances are of paramount relevance, and there is no operational reliability that the gases behave in the same manner in both mixtures and single-gas experiments. To our knowledge, only three reports currently investigate mixed-gas behavior in such membranes. A comparison between binary CO_2_ and single-gas permeances shows very different results within the limited data available. Whereas Yang et al. [26] report a decreasing permeance for binary mixtures, Rubner et al. [24] observed an increasing one and van Gestel. et al. [27] found no difference between mixtures and single-gas experiments. For this reason, we add further data and aim to explain the observed variations.

To our knowledge, no permeation data are available for organo-silica membranes operating in vacuum and atmospheric modes, making it difficult to compare the two. Only in other microporous systems were the vacuum permeation data entirely given [28]. It is assumed that low permeate pressures enhance the CO_2_ desorption kinetics and thus increase the permeance [29].

The present study aims to re-evaluate the permeation and separation behavior in organo-silica membranes, focusing on general and specific cases, using an organo-silica membrane fabricated in our lab. The investigation examines the CO_2_ and N_2_ permeance of the fabricated membrane in a single-gas operation. It evaluates the mass transport properties of each layer (macroporous support, mesoporous intermediate layer, and selective cover layer). We aim to correlate the permeance levels with the respective mass transport mechanism by examining the temperature trajectories. As these membranes operate to provide CO_2_ for processes like rWGS, we present the available data for various published organo-silica-based membranes in the Robeson-like plot for the respective temperatures.

After the single-gas examination, we conduct experiments on mixed-gas transport phenomena by testing the fabricated membrane in binary CO_2_/N_2_ mixtures and providing mixed-gas permeances as one of the first for this membrane. Finally, we also study how the mode of operation affects mass transport kinetics. This involves varying factors such as the CO_2_/N_2_ composition of the feed mixtures and the driving force (atmospheric or vacuum permeate pressure). We compare operation modes with permeation data to show why separation selectivity and separation factors are often confused, leading to misleading conclusions.

## 2. Materials and Methods

### 2.1. Membrane Formation

The support for membrane deposition was a polished macroporous α-alumina disk with a diameter of 39 mm, a thickness of 2.2 mm, and a pore size of 80 nm, purchased from Pervatech (PT) BV (the Netherlands). On top of this, a mesoporous γ-Al_2_O_3_ layer was coated by an aqueous sol–gel method using a boehmite (γ-AlO(OH)) sol. The synthesis has been described in the literature based on the original work of Yoldas [30]. The sol was mixed with a PVA solution (3.5 g PVA/100 mL water) in a ratio of 3:2 and then poured into a 50 mL Petri dish. We used a specially designed dip-coater, which moved clockwise through the Petri dish so that only the polished side of the support was immersed (holding time was 15 s). For this, the support was attached to a vacuum suction cup mounted on a rotating pendulum, and the dish with the sol/PVA mixture was positioned at the lowest point of the pendulum (see Figure S1). After coating, it was isothermally treated at 500 °C for 3 h with a pre-heating and post-cooling ramp of 1 °C/min.

On top of the obtained mesoporous γ-Al_2_O_3_ layer, a microporous organo-silica (Org-Sil) top layer was deposited by an alcoholic sol–gel method using the same pendulum dip-coater. Our synthesis and coating process was mainly based on previous work by Castricum et al. [20] and van Gestel et al. [27]. In summary, 16.66 mL of the membrane-forming precursor (1,2-bis(triethoxysilyl)-ethane (BTESE)) was mixed with 28.14 mL of ethanol (both thermo scientific chemicals), 0.63 mL of nitric acid (65 wt.%), and 4.57 mL of water at room temperature ([water]:[hydrolyzable ethoxy groups] = 1). The resulting mixture was heated to 60 °C under reflux conditions for 90 min, followed by natural cooling to room temperature. Then, the sol was diluted with 50 mL of ethanol and stored in a refrigerator (stock sol). Before the coating step, this stock sol was further diluted 20 times with ethanol to obtain defect-free films reliably. Finally, after dip-coating, the membrane sample was dried for ~1 h and thermally treated at 300 °C for 3 h under a N_2_ or air atmosphere to create different microporous structures [20,31,32,33]. A drawing of the resulting microstructure can be found elsewhere [5], and photographs of the membrane are shown in Figure S2.

### 2.2. Structural and Chemical Characterization

High-resolution scanning electron microscope images (SEM) were made using a Zeiss Supra 55 VP microscope with an acceleration voltage of 18 kV. The membrane disk was shock-frozen in liquid N_2_ for a few minutes to obtain high-quality images. Then, the membrane was cut into small 1 cm^2^ pieces using a side cutter, and the obtained piece was sputter-coated with a thin layer of gold to ensure electrical conductivity. Chemical characterization by ATR-FTIR and Raman spectroscopy, as well as X-ray diffraction (XRD) and thermo gravimetric (TG) analysis, was conducted in detail and are reported elsewhere [34].

### 2.3. Gas Permeation and Separation Experiments

Single-gas experiments were performed using a custom-made test cell designed to hold 39 mm disks, with the feed gas inlet and the retentate outlet on the upper side and the permeate outlet at the bottom side of the cell. A small opening at the top side allowed the application of a thermocouple non-invasive close to the membrane. The feed gas, CO_2_ or N_2_, was supplied by a gas bottle with a mounted pressure reducer, allowing measurements at pressures up to 6 bar. A Bronkhorst El Press pressure controller valve was installed in the retentate line. Pressure was measured in the feed p_f_ and the permeate p_p_ line. All the measurements were conducted with the same transmembrane pressure difference of 3.2 bar. Experiments with atmospheric pressure on the permeate side held a feed pressure of 4.2 bar; for experiments with a vacuum (70 mbar) on the permeate side, the feed pressure was lowered to 3.27 bar. No sweep gas was used. To quantify the mass flow of the permeating stream Ji, three Bronkhorst EL-Flow Prestige mass flow meters with different measurement ranges (0–1 g/h, 0–10 g/h, and 200 g/h) to reduce the measurement error were installed in the permeate line (error: ±0.5% of the measured value, ±0.1% of endpoint value). The membrane cell was placed in an oven to vary the temperature. To apply a lower pressure, a membrane pump (KNF LABORPORT N938.50) was installed in the permeate line. Before the permeation experiments, the membrane surface was sealed with a Viton O-Ring (i.d. 32 mm), resulting in an effective permeation area *A_m_* of 804 mm^2^. Due to the application of Viton, the testing temperature was limited to 200 °C; with the vacuum pump, a pressure of 70 mbar (absolute pressure) was obtained.

(1) $$Q_{i}=\frac{J_{i}}{A_{m}\cdot (p_{i,f}-p_{i,p})}=\frac{P_{i}}{d_{i}}$$

The permeance *Q* was calculated from the measured values according to Equation (1). Accordingly, the permeability *P_i_* as the intrinsic membrane kinetic was calculated by multiplying the permeance with the respective layer thickness *d_i_*.

(2) $$S_{CO_{2}/N_{2}}=\frac{Q_{CO_{2}}}{Q_{N_{2}}}$$

The permselectivity *S* was calculated as the ratio of the *CO*_2_ to *N*_2_ permeance as shown in Equation (2).

(3) $$\beta_{CO_{2}/N_{2}}=\frac{{\left(x_{CO_{2}}/x_{N_{2}}\right)}_{Permeate}}{{\left(x_{CO_{2}}/x_{N_{2}}\right)}_{Feed}}$$

In binary gas permeation experiments with *CO*_2_/*N*_2_ mixtures, Bronkhorst EL-Flow mass flow controllers were applied to create mixtures with 10, 25, and 50 vol % *CO*_2_. The composition of the permeate was analyzed with a micro gas chromatograph (µGC) (Agilent 490, Manchester, UK). The separation efficiency was evaluated using the separation factor β, defined in Equation (3) as the ratio of the mole fraction *x* of *CO*_2_ to *N*_2_ in the permeate compared to the same ratio in the feed:

(4) $$Q_{composite}={\left(\frac{d_{\alpha -Al_{2}O_{3}}}{P_{\alpha -Al_{2}O_{3}}}+\frac{d_{\gamma -Al_{2}O_{3}}}{P_{\gamma -Al_{2}O_{3}}}+\frac{d_{org-sil}}{P_{org-sil}}\right)}^{-1}$$

A resistance-in-series model was employed to calculate the consecutive mass transport resistances in the composite membrane [35]. 

First, the substrate’s permeance was measured (Equation (1)) as a stand-alone membrane, and with the respective membrane thickness *d_i_*, obtained from SEM images, the substrate permeability was calculated by the relevant summand of Equation (4). In the second step, the same substrate was coated with an γ-Al_2_O_3_ layer and combined with the information about the substrate and the γ-Al_2_O_3_ layer thickness; the permeability of this layer was calculated by Equation (4). This was repeated for the organo-silica layer.

## 3. Results

**Figure 3:**
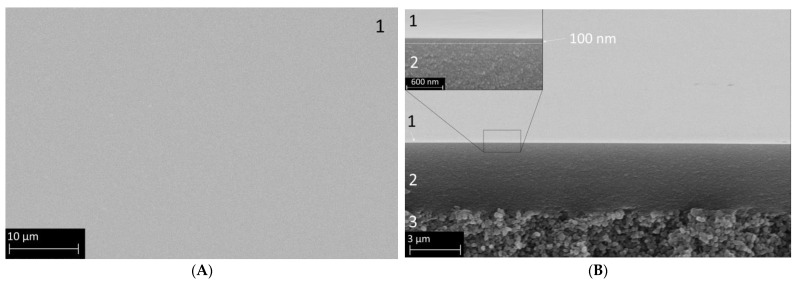
SEM secondary electron images of a composite membrane comprising the α-Al_2_O_3_ macroporous support (3), the mesoporous γ-Al_2_O_3_ layer (2), and the microporous organo-silica top layer fired in N_2_ (1). Panel (**A**) shows the surface view, and Panel (**B**) shows a cross-section with distinctive layer separation. The insert shows the membrane at a higher magnification to better visualize the organo-silica layer thickness.

Figure 3 displays the SEM images of a membrane sample prepared as described in the experimental section. The left panel (A) shows the defect-free surface, uniformly covered by the organo-silica layer. An edge view is presented in panel (B), where distinct macro-, meso-, and microporous parts are visible. Layer 3 represents the macroporous α-Al_2_O_3_ substrate, followed by a γ-Al_2_O_3_ layer with a thickness of 4 µm (layer 2), which is relatively large compared with the other publications in this field, possibly due to the higher amount of PVA in the coating liquid used here. However, as can be seen, the roughness of the α-Al_2_O_3_ substrate is, in this way, very effectively smoothened. In the insert of Figure 3B, the BTESE-derived functional organo-silica top layer 1 is also clearly visible. The estimated layer thickness is around 100 nm, which is comparable with values for similar membranes in the literature [20,27,36], and the surface looks very clean and defect-free.

**Figure 4:**
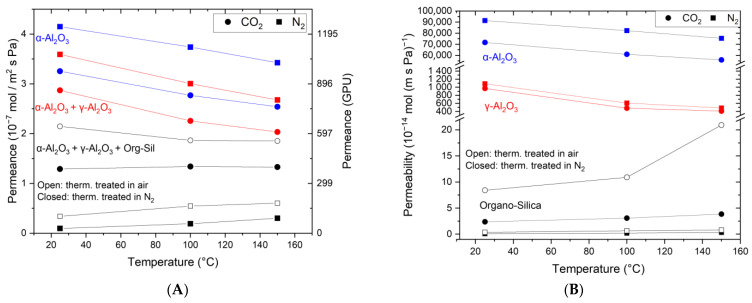
Temperature-dependent single-gas permeation through the α-Al_2_O_3_ support (blue), the support + γ-Al_2_O_3_ (red), and the selective cover layer made of organo-silica on top of the others (black). Panel (**A**) shows the measured permeance, and (**B**) the derived permeability.

Figure 4A shows the single-gas permeation results for CO_2_ and N_2_. The measurements include the stand-alone α-Al_2_O_3_ substrate (3), (3) + γ-Al_2_O_3_ layer (2), and (3 + 2) + organo-silica top layer, both the thermally treated version in N_2_ and air. The support (3) shows a lower permeance for CO_2_ (3.25 × 10^−7^ mol m^−2^ s^−1^ Pa^−1^) than for N_2_ (3.6 × 10^−7^ mol m^−2^ s^−1^ Pa^−1^) at ambient temperature. The permeances for both components decrease with increasing temperature according to the Knudsen theory. For all the temperatures, the observed permselectivity is around 0.8, which equals the theoretical Knudsen selectivity. The same behavior is also observed after adding the γ-Al_2_O_3_ layer. However, the permeance is lower (CO_2_: 2.87 × 10^−7^ mol m^−2^ s^−1^ Pa^−1^; N_2_: 3.6 × 10^−7^ mol m^−2^ s^−1^ Pa^−1^) as an additional resistance is introduced. Such pores could enhance the surface diffusion mechanism for CO_2_ as described in reference [37].

Coating with the organo-silica layer leads to essential changes in the gas permeation behavior. As can be seen, the CO_2_ permeance exceeds the N_2_ permeance, albeit at a lower level. The permeance values are reduced to 1.3 × 10^−7^ mol m^−2^ s^−1^ Pa^−1^ for CO_2_ and 0.1 × 10^−7^ mol m^−2^ s^−1^ Pa^−1^ for N_2_ for the organo-silica thermally treated in N_2_. Again, the same trend is observed in the air-treated one, but the permeance levels are higher for both components. Notably, the permeance of N_2_ is now much lower than that of CO_2_, demonstrating that the membrane became CO_2_-affine and suitable for CO_2_ separation applications. We assume that N_2_ molecules can only diffuse through the free pore volume in the membrane layer. In contrast, the CO_2_ molecules can use an additional diffusion path along the inner pore surface, making surface diffusion the prominent mass transport mechanism. The obtained CO_2_/N_2_ permselectivity of 13 for organo-silica treated in N_2_ is around 15 times higher than that for a non-selective Knudsen membrane. When treated in air the CO_2_/N_2_ permselectivity of 6.3 is around 8 times higher than for a non-selective Knudsen membrane.

Equally interesting are also the trends observed upon increasing the testing temperature. For N_2_, a slight but measurable increase is obtained going from room temperature to 100 °C and further to 150 °C. This suggests an activated gas transport mechanism typical for relatively narrow microporous structures (pore diameter < 1 nm) and the absence of a substantial Knudsen transport contribution [38]. Remarkably, the CO_2_ permeance remains constant, which is unexpected considering the lower adsorptive affinity for CO_2_ but already observed elsewhere [26,27,29,39]. In these tests, activated transport and surface diffusion contribute equally to the overall CO_2_ transport through the membrane and neutralize each other. From these results, it is also clear that the permselectivity of the membrane is negatively affected by these temperature-dependent trends. An increase in the temperature to 100 °C and further to 150 °C leads to a lowering of the permselectivity to 7 and 4.3 for the membrane treated in N_2_, and for the membrane treated in air, the selectivity decreases from 3.4 to 3. Both cases are still larger than the Knudsen value of 0.8.

In a multi-layered system consisting of a macroporous support, mesoporous layers, and a microporous selective top layer as described here, the measured permeance and permselectivity values reflect the mass transfer resistance in the permeation through the membrane, which is expressed in terms of flux normalized per unit of pressure and area (mol/m^2^ s Pa). To facilitate comparison, the permeation data in Figure 4A were then normalized per unit of thickness for each part of the membrane, resulting in the intrinsic permeability values, see Equation (4). As expected, the results summarized in Figure 4B show that the macroporous α-Al_2_O_3_ support (thickness 2.2 mm) exhibits by far the highest permeability and, thus, the lowest resistance in the membrane structure. When we consider the mesoporous γ-Al_2_O_3_ layer as a stand-alone membrane (thickness 4 μm), the permeability is much lower, and a CO_2_/N_2_ permselectivity of 0.9 is obtained. We can see further a similar trend for the stand-alone organo-silica layer thermally treated in N_2_, which exhibits a permselectivity of ca. 23 at 20 °C, decreasing to 15 and 11 at 100 and 150 °C, respectively. Notably, the selectivity increases for the in-air treated membrane from 23 at 20 °C to 16 at 100 °C to 26 at 150 °C. The differences between the theoretically calculated and measured values suggest faster CO_2_ permeating kinetics in the organo-silica layer of the membrane structure. However, this is negatively compensated by the dominating Knudsen diffusion contribution of the support structure, where N_2_ shows faster kinetics. Remarkably, in the single organo-silica layer, increased permeance of CO_2_ is seen for the membrane treated in air. This phenomenon is not seen in the composites as the slower CO_2_ kinetics in the support structure overlay and dominate the overall kinetics. To evaluate the quality and separation performance of the membrane’s top layer, the transport kinetics of the underlying layers must also be analyzed, as the entire multi-layer membrane has a lower selectivity than the top layer.

**Figure 5:**
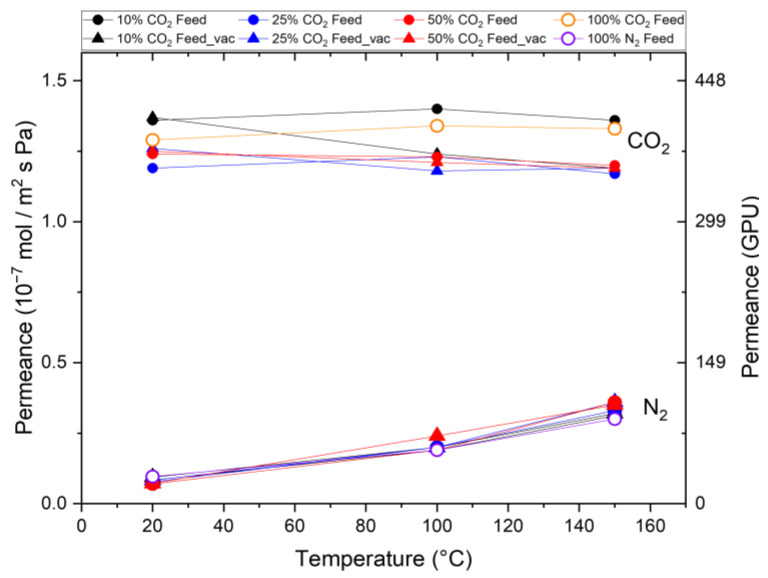
Mixed-gas permeation through Org-Sil membrane treated in N_2_. Permeances of CO_2_ and N_2_ for different gas compositions (10%, 25%, and 50% CO_2_) and permeate pressures (atmospheric (○) and 70 mbar (△)) at different temperatures. Single-gas measurements (100% CO_2_ and 100% N_2_ as reference). The transmembrane pressure is kept constant at ∆*p* = 3.2 bar.

In the next series of experiments, CO_2_ and N_2_ are applied as a mixture to the membrane treated in N_2_. A transmembrane pressure of 3.2 bar was applied. To quantify the influence of CO_2_ desorption on the permeation behavior, the pressure on the permeate side was set to 70 mbar. Figure 5 shows the observed CO_2_ and N_2_ permeances for different feed mixtures with 10 vol%, 25 vol%, and 50 vol% CO_2_ at both pressure levels as a function of the testing temperature. It can be seen that the N_2_ permeance increases from room temperature to 100 °C and further to 150 °C for each feed composition by analogy with the single-gas permeation tests (100% N_2_). This confirms that N_2_ exhibits activated diffusion in the membrane’s micropores in a mixed-gas situation. In contrast, a constant CO_2_ permeation with increasing temperature in the single-gas permeation tests was observed in all the mixed-gas experiments regardless of the composition of the feed. This indicates once again that activated permeation and surface diffusion balance each other out or that the influence of the support structure overlays the temperature increase.

The trends observed in the mixed-gas results are consistent with those from the single-gas experiments, including CO_2_/N_2_ selectivity, which shows agreement with the permselectivity values calculated from the single-gas tests. For example, an identical selectivity of 13.5 is achieved at room temperature, which decreases to 3–4 for the test at 150 °C. This indicates that the widely accepted mechanism of pore blocking by CO_2_ and consequent reduction in N_2_ permeation does not occur. If such a pore-blocking mechanism were to happen when both gases are applied as a mixture to a microporous membrane, the N_2_ permeance would decrease in the mixed-gas situation.

Figure 5 also shows the results of alternative permeation measurements for the case where the pressure on the permeate side is reduced to 70 mbar. We do not observe differences from the previous pressure-driven experiments with atmospheric pressure on the permeate side, which shows that the CO_2_ and N_2_ permeation is not dependent on the pressure on the permeate side. Furthermore, as for all the measuring points, a different CO_2_ partial pressure on the feed side was applied, and the same permeance was observed. A pressure dependence on the permeance can be ruled out.

**Figure 6:**
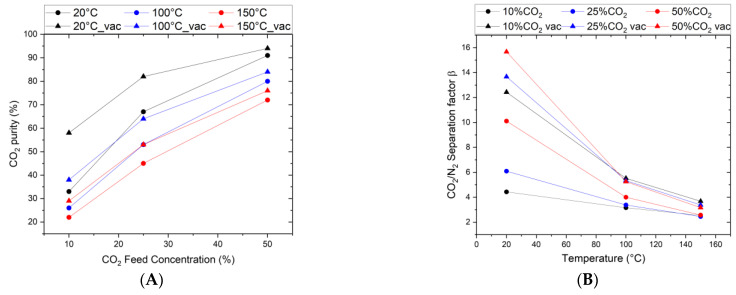
CO_2_/N_2_ gas mixture separation through Org-Sil membrane. (**A**) CO_2_ purity in permeate as a function of the CO_2_ Feed Composition, (**B**) CO_2_/N_2_ separation factor as a function of temperature for different feed compositions (10, 25, and 50 vol% CO_2_, Δ denotes permeate vacuum). The transmembrane pressure is kept constant at ∆*p* = 3.2 bar.

In studying the permeation and separation properties of the top layer, the effective separation factor is an important parameter. However, descriptions of permeation and separation in microporous membranes covering this aspect are hard to find. The separation factor is a characteristic parameter that describes the separation efficiency of a binary mixture. It measures the enrichment of a gas component (e.g., CO_2_) after it has passed the membrane. Thus, it is, together with the permeance, the main factor determining the practical applicability of the membrane. Figure 6A shows the effect of feed composition, pressure, and temperature on the separation factor and the related CO_2_ enrichment from the feed gas. As can be seen, the higher the CO_2_/N_2_ ratio in the feed, the higher the separation efficiency. When comparing atmospheric pressure with reduced pressure at the permeate side, the separation factor shifts to higher values when a vacuum is applied on the permeate side. As shown in Figure 6B, this effect is pronounced for the room temperature results with, e.g., a separation factor increase from 10 to 16 for a 50/50% CO_2_/N_2_ feed mixture. Calculated in terms of CO_2_ purity in the permeate flow, this results in very high values of 90–95%. Following the single-gas permselectivity, the separation factor decreases continuously with increasing temperature, converging at 150 °C to values of 2 to 4, which the increased N_2_ kinetics and a temperature constant CO_2_ permeance can explain.

## 4. Discussion

**Figure 7:**
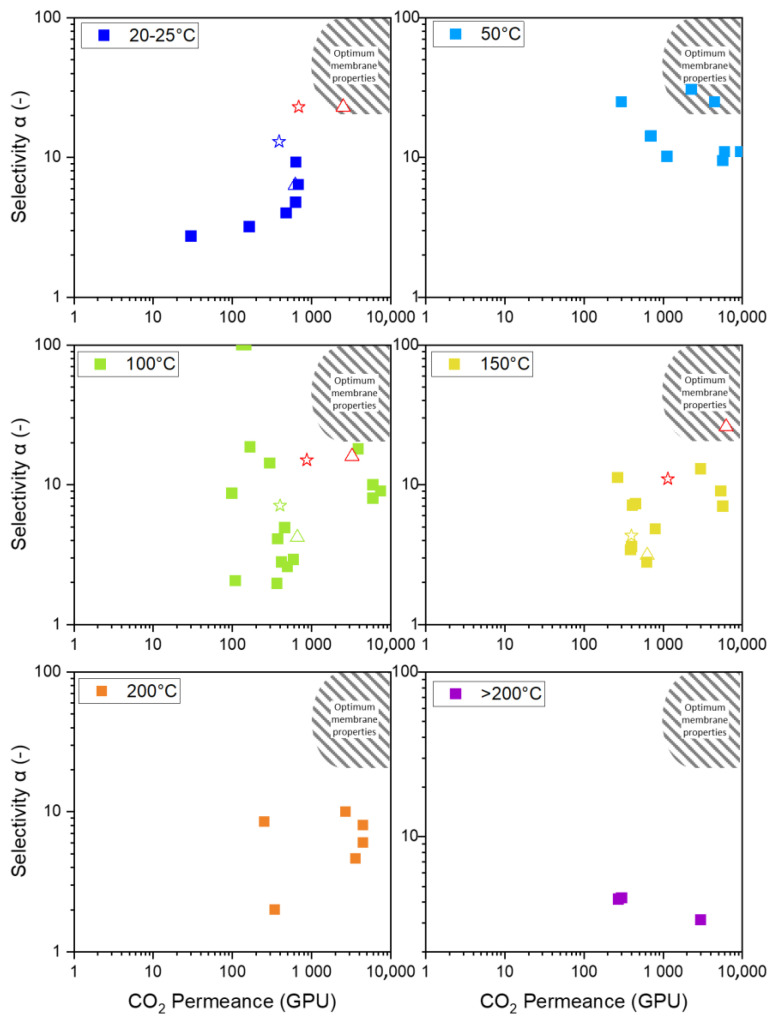
Display of available BTESE-derived permeance and selectivity in a Robeson-like plot for temperatures from 20 °C to over 200 °C. Each square □ represents a membrane from the references. The star ☆ indicates the membrane presented in this study, treated in N_2_, while the triangle △ represents the membrane treated in air. The red stars and triangles correspond to theoretical stand-alone membranes. Data from references [24,25,26,27,29,31,36,39,40,41,42].

Figure 7 displays a Robeson-like plot of various organo-silica and primarily BTESE-derived membranes. As the future interest lies in the application at higher temperatures, the available data for temperatures up to >200 °C are also shown. Although no data at 50 °C are presented, this temperature panel is included as the membrane reported by Guo et al. [25] at 50 °C is the only one that reaches the envisioned area of optimum membrane properties. As the temperature increases, even the best-performing membrane leaves this area.

The membrane discussed in this article is indicated by the star (in N_2_) and the triangle (in air). The membrane treated in N_2_ is located at the arrow’s starting point, and the arrowhead points to the membrane treated in air. The initial approach was to modify the pore structure to increase the number of pores while keeping the pore size constant, as illustrated in Figure 2 by the green arrow. However, this approach did not produce the intended result, suggesting increased pore size. This behavior was also observed by Ibrahim et al., who describe the increased permeance by an increase in pore size. Thermal treatment in air leads to a higher degree of decomposition of organic ethoxide groups [33].

Comparing the membranes at different temperatures is difficult for two major reasons. Firstly, all membranes have different properties, as shown in Figure 2. The microstructure of the membranes has (i) different porosity for CO_2_ (green arrow), (ii) different pore sizes (blue arrow), and (iii) a varying degree of active sites that participate in surface diffusion (red arrow). However, the pore structure and number are often undisclosed along the permeation data. This is either because of laborious investigation procedures or influences on the pore structure, which are various and usually not easy to control. Membranes fabricated similarly often vary in their permeation data because of varying influences. The discrepancies can be attributed to the specific fabrication processes, including the water/silica and acid/silica ratio, reaction time, reaction temperature, stirring, sol aging (dilution and particle growth), drying conditions (humidity, duration, temperature, and defects), sintering (temperature, time, atmosphere, and defects), coating technique (dilution, coating times, and airborne particles), and membrane aging (measurement immediately after fabrication or after a storage period).

Secondly, the practice of reporting the permeation data as the permeance of the composite and not as the permeability of the respective layer complicates the comparability. In Figure 7, organo-silica-derived membranes on different support structures are shown, making it difficult to compare the resulting permeation data. In Figure 4B, we presented permeability data for our membranes and noticed a different temperature trajectory for the composite than for the single layers. This is possibly due to differences in the mass transport phenomena of the mesoporous support structure, which affects the behavior of the separation layer. We calculated the permeance of a theoretical single layer and placed it in the Robeson-like plot to illustrate the impact of the support. The red squares in the panels for 20 °C, 100 °C, and 150 °C represent the theoretical single-layer permeances for the fabricated membrane treated in N_2_ and air. This demonstrates that the air-treated membrane now meets the necessary permeation properties for economic application. We understand that some support structure is needed, and we believe that with material optimization, some of the membranes currently not in the optimum area will reach that point. We want to highlight how the mesoporous support becomes more influential at higher temperatures because the dominating Knudsen diffusion decreases its flux. Therefore, high flux supports can help make the application economically viable at high temperatures [43].

Nevertheless, we tried to explain the different permeation and selectivity levels in the published data and how apparent contradictions can be resolved. Examining the temperature trajectories of different gas permeances offers insights into the pore structure. Three different cases are observed. Firstly, Castricum et al. and Kanezashi et al. report decreasing permeances for CO_2_ with increasing temperature [31,41]. Secondly, Grekou et al. [24,29] and Rubner et al. demonstrate the opposite trend, showing an increase in CO_2_ permeance with rising temperature [24,29]. Thirdly, Van Gestel et al. and Yang et al., as well as our work, observe no changes in permeances with increasing temperatures [26,27]. Three distinct mass transport mechanisms—Knudsen diffusion, surface diffusion, and activated permeation—coexist depending on the number and size of pores, participating in mass transport in varying ratios. Higher proportions of Knudsen diffusion and surface diffusion result in higher permeances but at a decreasing trend with increasing temperature. Therefore, in the first case, it can be concluded that Castricum et al. [41] and Kanezashi et al. [31] produced membranes with many micro- and mesopores. Membranes with activated diffusion as the predominating mass transport mechanism give low permeances but show an increasing trend with increasing temperature. Therefore, in the second case, the membranes reported by Grekou et al. [29] and Rubner et al. [24] indicate a microstructure with a high proportion of ultramicropores. In the third case, membranes with a pore structure in between can have mass transport mechanisms that offset each other, resulting in constant permeances with increasing temperature. The membranes studied by van Gestel et al. [27], Yang et al. [26], and those investigated in this work are likely to have structures with micro- and ultramicropores. Therefore, it is concluded that for a membrane design, only microstructures with pores below 0.7 nm are suitable, as only these show large permeances with good selectivity. The problem with decreasing selectivity at higher temperatures is assumed to be overcome by providing active sites for surface diffusion at higher temperatures.

Using permeances obtained from single-gas measurements for mixed-gas applications can cause problems since the interactions of mixed gases can alter their behavior. Membranes with a large portion of ultramicropores can enable interactions between the slow and fast permeating compounds by a pore-blocking or condensation mechanism [44]. Yang et al. showed a decrease in CO_2_ in the mixture compared to the single gases and accounted for some hindrance as its cause, as reported in zeolites [13,26]. Rubner et al. even observed increased CO_2_ permeances in the mixture compared to the single-gas results, indicating that the non-adsorbing gas enhances the mass transport of the adsorbing gas. Our results fall within the range of reported data, showing no deviations from the single-gas results, indicating that no interactions occur in micropores.

Since most publications do not disclose mixed-gas permeances, they assess their mixed-gas results based on the separation factor and compare them to the single-gas permselectivity. If the separation factor is lower than the permselectivity, some hindrance or blockage is attributed to the decreased selectivity. This approach can lead to incorrect conclusions since the separation factor depends on kinetics (permeance) and the driving force (partial pressure difference) governing mass transport.

As illustrated in Figure 6B, the same membrane exhibits variations in its separation factor from 5 to 10 by merely changing the feed composition (10% to 50%). Additionally, for the same composition (10% CO_2_), applying a vacuum on the permeate side causes the separation factor to change from 4 to 13. However, the permselectivity remains constant throughout (see Figure 5). For a proper comparison, mixed-gas permeances are necessary.

Operating mixed-gas separations on the permeate side under vacuum conditions yields higher separation factors. Contrary to the proposition by Grekou et al. [29] that the higher separation factors are caused by the enhanced desorption kinetics of the adsorbing component under lower pressure, our observations indicate otherwise. If the adsorption were truly enhanced, the permeances for different operation modes (atmospheric vs. vacuum) would show variation. Operating the membrane with a vacuum increases the driving force (partial pressure difference) for the faster-permeating component, leading to higher fluxes and higher permeate concentrations at low pressures [45].

## 5. Conclusions

In this study, we investigated ceramic microporous CO_2_-philic membranes. We analyzed the transport behavior of CO_2_ and N_2_ focusing on the possible future implementation of such a membrane in a CO_2_ separation process at elevated temperatures.

The investigated membrane shows a constant CO_2_ permeance with increasing temperature and an even increasing permeability for the membrane thermally treated in air. This shows that focusing on permeances overlooks the actual transport behavior of the fabricated organo-silica layer, as the influence of the support structure is non-neglectable. A single-gas separation efficiency derived from permeances at 150 °C yields a selectivity of 4.3. In contrast, the same membrane has a selectivity of 11 when single-layer permeabilities are used as mass transport kinetics. It is important to note that the Knudsen behavior of the support structure often masks the properties of the selective cover layer. Only conclusions based on permeabilities are meaningful when evaluating different organo-silica fabrication methods.

As all membranes are aimed to perform in mixed-gas applications, single-gas kinetics must be compared to mixed-gas behavior to derive the relevant kinetics and separation performance. The presented membrane shows the same kinetics and selectivities as in single-gas experiments. The elsewhere reported separation performance increases based on a pore-blocking mechanism can not be confirmed as the anticipated decrease in N_2_ permeance was not observed.

However, the separation factor based on operation conditions showed that the membrane is pressure ratio limited, and the separation factor increases with increasing pressure ratios (Figure 6A,B). This is achieved by increasing the partial pressure in the feed or by lowering the partial pressure in the permeate via a vacuum. An enhanced CO_2_ desorption kinetic caused by permeate vacuums was also not observed, as the CO_2_ permeance stayed constant for all the operation modes.

This study introduces an extension of the Robeson-like plot for different temperatures, including membrane data reported in the literature and data from the present study. Only one membrane at 50 °C reaches the optimum membrane properties across all the available data. Until now, no organo-silica-based membrane has reached the properties for economic application above 50 °C.

From temperature trajectories introduced into the Robeson plot, we conclude that only membranes with micropores in the range for surface diffusion can reach the envisioned area of optimum membrane properties. This is especially important for high-temperature applications where the surface affinity is declining. Combining micropores with surfaces that offer adsorption sites that enable surface diffusion at higher temperatures is very promising [5,34].

## Data Availability

The data that support the findings of this study are available from the corresponding author upon request.

## Supplementary Materials

The following supporting information can be downloaded at: https://www.mdpi.com/article/10.3390/membranes15030083/s1, Figure S1: Rotary dip coating setup. The top left picture shows the pendulum moved by a motor in a rotary motion. At the end of the pendulum is a substrate mounted by suction. This is moved through a petri dish filled with the coating sol. The right panel shows a schematic drawing of the laboratory setup. The bottom left picture shows a magnification of the critical point where the coated substrate leaves the sol and a drop forms at the rim; Figure S2: Photograph of the BTESE-coated Substrate in the Laboratory on the left. The same BTESE membrane inside the permeation cell on the right.
