# Supplementary material for: Study on Organo-Silica-Derived Membranes Using a Robeson-like Plot
Source: Membranes (Basel). 2025 Mar 5;15(3):83. doi: 10.3390/membranes15030083 (PMC11943757; doi:10.3390/membranes15030083)
Supplement: Supplementary file 1 [file membranes-15-00083-s001.zip › membranes-3480868-supplementary.pdf]

Supplementary Information (SI)

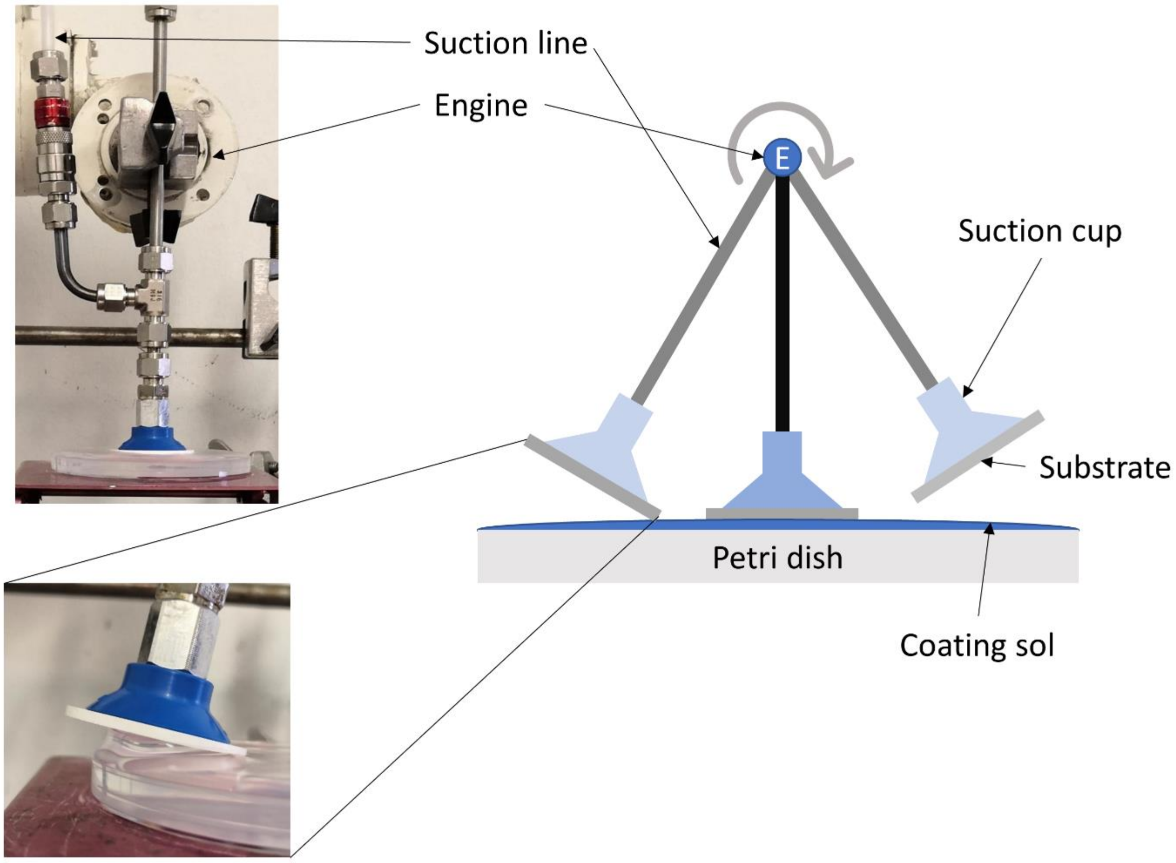

Figure S1. Rotary dip coating setup. The top left picture shows the pendulum moved by a motor in a rotary motion. At the end of the pendulum is a substrate mounted by suction. This is moved through a petri dish filled with the coating sol. The right panel shows a schematic drawing of the laboratory setup. The bottom left picture shows a magnification of the critical point where the coated substrate leaves the sol and a drop forms at the rim.

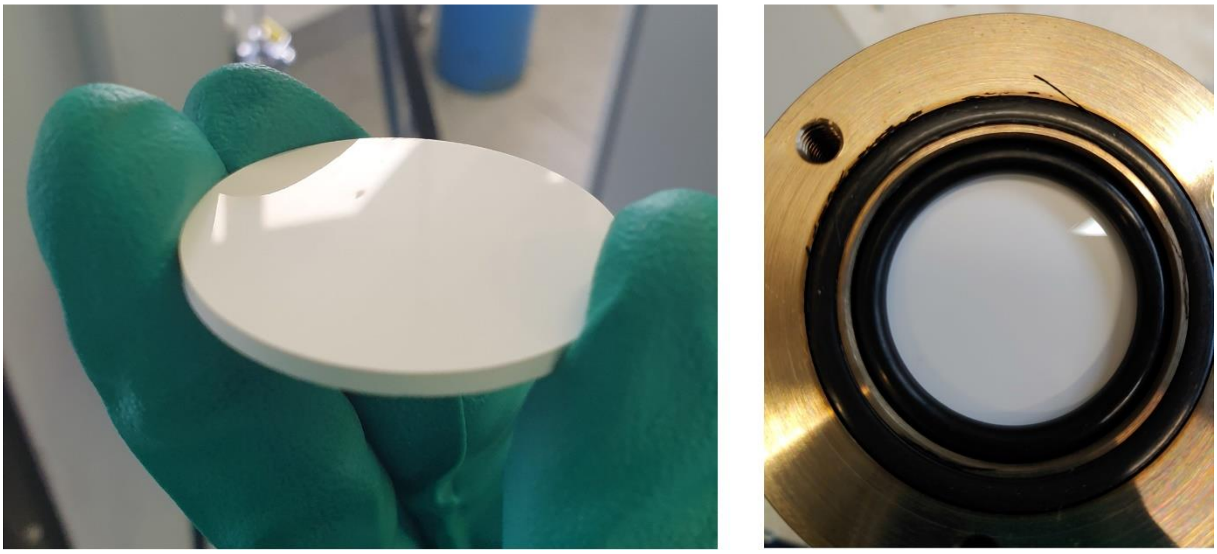

Figure S2. Photograph of the BTESE-coated Substrate in the Laboratory on the left. The same BTESE membrane inside the permeation cell on the right.
